# *catena*-Poly[[[{1-[(*E*)-phenyl(pyridin-2-yl-κ*N*)methylidene]semicarbazidato-κ^2^ *N* ^1^,*O*}copper(II)]-μ-dicyanamido-κ^2^ *N* ^1^:*N* ^5^] monohydrate]

**DOI:** 10.1107/S1600536812035283

**Published:** 2012-08-15

**Authors:** Roji J. Kunnath, M.R. Prathapachandra Kurup, Seik Weng Ng

**Affiliations:** a Department of Applied Chemistry, Cochin University of Science and Technology, Kochi 682 022, India; b Department of Chemistry, University of Malaya, 50603 Kuala Lumpur, Malaysia; c Chemistry Department, King Abdulaziz University, PO Box 80203 Jeddah, Saudi Arabia

## Abstract

The Cu^II^ atoms in the title coordination polymer, {[Cu(C_13_H_11_N_4_O)(C_2_N_3_)]·H_2_O}_*n*_, are *N*,*N*′,*O*-chelated by the deprotonated Schiff base ligands, and adjacent metal atoms are bridged by the dicyanamide ions, generating a polymeric chain that propagates along the *b* axis. The two independent metal atoms show a square-pyramidal N_4_O coordination. The two independent water mol­ecules are disordered over two positions; each water mol­ecule is a hydrogen-bond donor to a carbonyl O atom. Weak N—H⋯N hydrogen bonding is also observed.

## Related literature

For the synthesis of the Schiff base ligand, see: de Lima *et al.* (2008 ▶). For a related copper(II) derivative, see: Peŕez-Rebolledo *et al.* (2006 ▶).
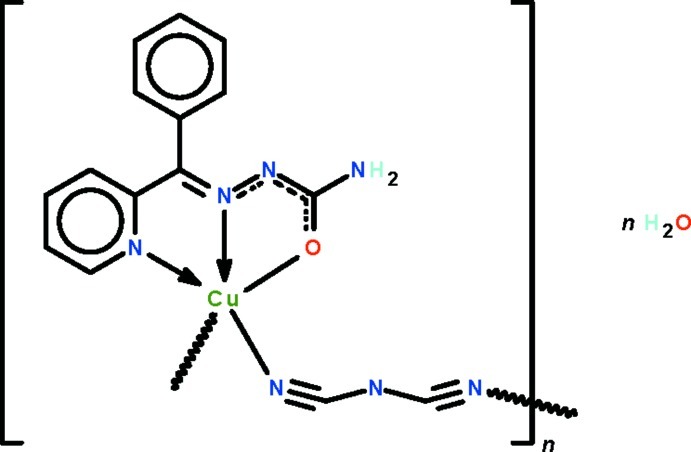


## Experimental

### 

#### Crystal data

[Cu(C_13_H_11_N_4_O)(C_2_N_3_)]·H_2_O
*M* *_r_* = 386.86
Orthorhombic, 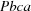

*a* = 12.3996 (2) Å
*b* = 21.0115 (4) Å
*c* = 26.7059 (5) Å
*V* = 6957.8 (2) Å^3^
*Z* = 16
Mo *K*α radiation
μ = 1.28 mm^−1^
*T* = 293 K
0.40 × 0.30 × 0.20 mm

#### Data collection

Bruker Kappa APEXII diffractometer
Absorption correction: multi-scan (*SADABS*; Sheldrick, 1996 ▶) *T*_min_ = 0.629, *T*_max_ = 0.784
110924 measured reflections
7982 independent reflections
5027 reflections with *I* > 2σ(*I*)
*R*_int_ = 0.054

#### Refinement

*R*[*F* ^2^ > 2σ(*F* ^2^)] = 0.050
*wR*(*F* ^2^) = 0.166
*S* = 1.12
7982 reflections
457 parameters
12 restraints
H-atom parameters constrained
Δρ_max_ = 0.80 e Å^−3^
Δρ_min_ = −0.46 e Å^−3^

### 

Data collection: *APEX2* (Bruker, 2010 ▶); cell refinement: *SAINT* (Bruker, 2010 ▶); data reduction: *SAINT*; program(s) used to solve structure: *SHELXS97* (Sheldrick, 2008 ▶); program(s) used to refine structure: *SHELXL97* (Sheldrick, 2008 ▶); molecular graphics: *X-SEED* (Barbour, 2001 ▶); software used to prepare material for publication: *publCIF* (Westrip, 2010 ▶).

## Supplementary Material

Crystal structure: contains datablock(s) global, I. DOI: 10.1107/S1600536812035283/xu5601sup1.cif

Structure factors: contains datablock(s) I. DOI: 10.1107/S1600536812035283/xu5601Isup2.hkl

Additional supplementary materials:  crystallographic information; 3D view; checkCIF report

## Figures and Tables

**Table 1:** Hydrogen-bond geometry (Å, °)

*D*—H⋯*A*	*D*—H	H⋯*A*	*D*⋯*A*	*D*—H⋯*A*
N4—H42⋯N7^i^	0.88	2.13	3.006 (5)	176
N8—H82⋯N3^ii^	0.88	2.15	3.025 (5)	179
O1w—H1w1⋯O1	0.84	2.05	2.88 (2)	169
O2w—H2w1⋯O2	0.84	2.34	3.151 (19)	161

Symmetry codes: (i) 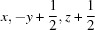; (ii) 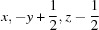.

**Fig. 1.**
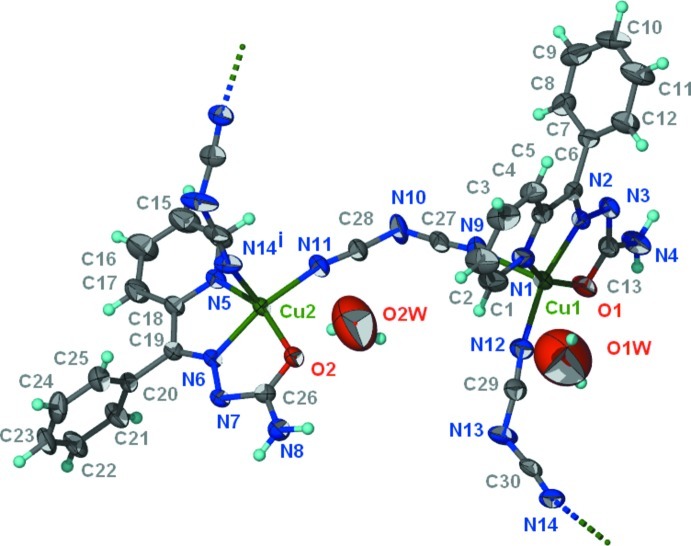
Thermal ellipsoid plot (Barbour, 2001) of a portion of the chain structure of [Cu(C2N3)(C13H11N4O).H2O]n at the 50% probability level; hydrogen atoms are drawn as spheres of arbitrary radius. The disorder in the water molecules is not shown.

**Fig. 2.**
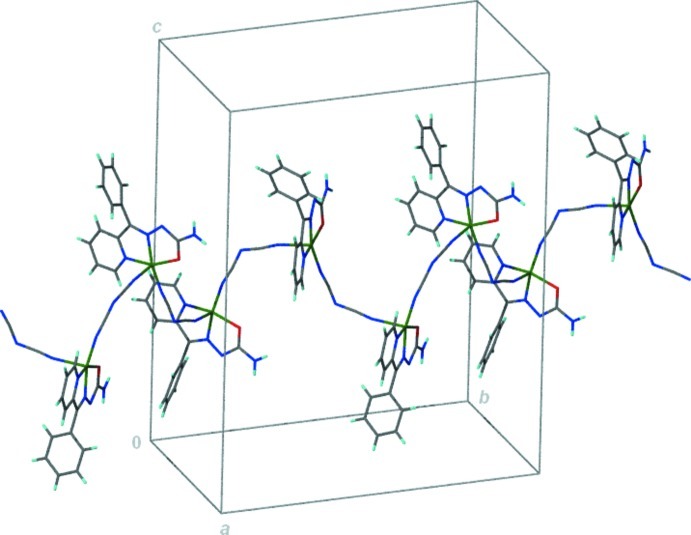
Dicyanamide-bridged chain structure. Water molecules are not shown.

## supplementary crystallographic information

### Comment

2-Benzoylpyridine semicarbazone (de Lima *et al.*, 2008) is a Schiff base that is capable of *N*,*N*',*O*-chelation to transition metal ions. This feature has been documented a copper(II) dichloride adduct; in this, the Schiff base exists as a neutral molecule (Peŕez-Rebolledo *et al.*, 2006). However, the Cu^II^ atom in the coordination polymer, [Cu(C_2_N_3_)(C_13_H_11_N_4_O)^.^H_2_O]*_n_* (I), is *N*,*N*',*O*-chelated instead by the deprotonated Schiff base (Fig. 1). Adjacent metal atoms are bridged by the dicyanamide ion to generate a chain that propagates along the *b* axis of the orthorhombic unit cell (Fig. 2). The two independent metal atoms show square pyramidal coordination. The two independent water molecules are disordered over two positions; each water molecule is a hydrogen-bond donor to a carbonyl O atom.

### Experimental

A methanol solution (20 ml) of 2-benzoylpyridine semicarbazone (0.240 g,1 mmol) (de Lima *et al.*, 2008), copper acetate monohydrate (0.199 g, 1 mmol) and sodium dicyanamide (0.089 g, 1 mmol) was heated for 5 h. The dark green solid was collected and recrystallized from methanol.

### Refinement

Carbon-bound H-atoms were placed in calculated positions (C–H 0.93 Å) and were included in the refinement in the riding model approximation, with *U*(H) set to 1.2*U*(C). The amino H-atoms were similarly treated (N–H 0.88 Å) and their temperature factors tied by a factor of 1.2 times.

Omitted owing interference from the beam stop were (2 1 0), (0 0 2), (1 1 2), (1 1 4) and (1 2 1).

The presence of water was indicated by an infrared spectral measurement. The two independent water molecules are both disordered over two positions; the occupancy could not be refined, and was assumed as a 1:1 type of disorder. For one molecule, the disorder is such that two components are separated by about 2 Å, so that one hydrogen atom should be midway between two oxygen atoms. For the other, the two are separated by about 1 Å, so that one hydrogen atom should be occupying the site of the other oxygen atom. For both, hydrogen atoms were positioned on only one component oxygen atom so that each water molecule forms only one hydrogen bond. Furthermore, the hydrogen atoms were given full occupancy, *i.e.*, hydrogen atoms were not placed on those atoms that do not engage in hydrogen bonding. The temperature factors of the primed atoms were set to those of the unprimed ones, and the anisotropic temperature factors were tightly restrained to be nearly isotropic.

### Crystal data

**Table:**

[Cu(C_13_H_11_N_4_O)(C_2_N_3_)]·H_2_O	*F*(000) = 3152
*M**_r_* = 386.86	*D*_x_ = 1.477 Mg m^−^^3^
Orthorhombic, *P**b**c**a*	Mo *K*α radiation, λ = 0.71073 Å
Hall symbol: -P 2ac 2ab	Cell parameters from 9967 reflections
*a* = 12.3996 (2) Å	θ = 2.7–26.5°
*b* = 21.0115 (4) Å	µ = 1.28 mm^−^^1^
*c* = 26.7059 (5) Å	*T* = 293 K
*V* = 6957.8 (2) Å^3^	Prim, green
*Z* = 16	0.40 × 0.30 × 0.20 mm

### Data collection

**Table:**

Bruker Kappa APEXII diffractometer	7982 independent reflections
Radiation source: fine-focus sealed tube	5027 reflections with *I* > 2σ(*I*)
Graphite monochromator	*R*_int_ = 0.054
ω scans	θ_max_ = 27.5°, θ_min_ = 1.9°
Absorption correction: multi-scan (*SADABS*; Sheldrick, 1996)	*h* = −16→14
*T*_min_ = 0.629, *T*_max_ = 0.784	*k* = −27→27
110924 measured reflections	*l* = −34→34

### Refinement

**Table:**

Refinement on *F*^2^	Primary atom site location: structure-invariant direct methods
Least-squares matrix: full	Secondary atom site location: difference Fourier map
*R*[*F*^2^ > 2σ(*F*^2^)] = 0.050	Hydrogen site location: inferred from neighbouring sites
*wR*(*F*^2^) = 0.166	H-atom parameters constrained
*S* = 1.12	*w* = 1/[σ^2^(*F*_o_^2^) + (0.0668*P*)^2^ + 9.8211*P*] where *P* = (*F*_o_^2^ + 2*F*_c_^2^)/3
7982 reflections	(Δ/σ)_max_ = 0.001
457 parameters	Δρ_max_ = 0.80 e Å^−^^3^
12 restraints	Δρ_min_ = −0.46 e Å^−^^3^

### Fractional atomic coordinates and isotropic or equivalent isotropic displacement parameters (Å^2^)

**Table:**

	*x*	*y*	*z*	*U*_iso_*/*U*_eq_	Occ. (<1)
Cu1	0.74889 (4)	0.32160 (2)	0.591725 (16)	0.03950 (15)	
Cu2	0.51236 (4)	0.06952 (2)	0.406562 (15)	0.03717 (15)	
O1	0.6417 (2)	0.38125 (13)	0.61888 (10)	0.0541 (8)	
O2	0.6166 (2)	0.13248 (12)	0.38040 (10)	0.0489 (7)	
O1W	0.4729 (16)	0.4332 (10)	0.5571 (8)	0.269 (7)	0.50
H1W1	0.5266	0.4228	0.5745	0.404*	
H1W2	0.4808	0.4710	0.5475	0.404*	
O2W	0.8427 (16)	0.1463 (9)	0.4332 (7)	0.234 (6)	0.50
H2W1	0.7879	0.1336	0.4177	0.280*	
H2W2	0.8367	0.1858	0.4373	0.280*	
O1W'	0.4691 (17)	0.5292 (11)	0.5296 (8)	0.269 (7)	0.50
O2W'	0.7929 (19)	0.1888 (10)	0.4402 (8)	0.234 (6)	0.50
N1	0.8834 (3)	0.26882 (16)	0.58984 (12)	0.0469 (8)	
N2	0.7736 (3)	0.31179 (14)	0.66329 (11)	0.0362 (7)	
N3	0.7020 (3)	0.33965 (15)	0.69487 (11)	0.0419 (7)	
N4	0.5629 (3)	0.41008 (19)	0.69133 (14)	0.0644 (11)	
H41	0.5184	0.4351	0.6749	0.077*	
H42	0.5586	0.4073	0.7241	0.077*	
N5	0.3807 (3)	0.01320 (15)	0.40768 (10)	0.0396 (7)	
N6	0.4882 (2)	0.06112 (14)	0.33509 (11)	0.0358 (7)	
N7	0.5555 (3)	0.09307 (15)	0.30382 (11)	0.0394 (7)	
N8	0.6909 (3)	0.16608 (17)	0.30793 (13)	0.0555 (9)	
H81	0.7353	0.1909	0.3246	0.067*	
H82	0.6936	0.1649	0.2750	0.067*	
N9	0.6399 (4)	0.2399 (2)	0.58076 (14)	0.0683 (12)	
N10	0.5250 (4)	0.1525 (2)	0.55530 (14)	0.0735 (13)	
N11	0.5158 (3)	0.09809 (17)	0.47568 (12)	0.0497 (8)	
N12	0.7496 (3)	0.35269 (18)	0.52272 (13)	0.0509 (9)	
N13	0.7622 (4)	0.3993 (2)	0.43995 (14)	0.0799 (15)	
N14	0.8717 (3)	0.48903 (18)	0.41363 (13)	0.0539 (9)	
C1	0.9380 (4)	0.2496 (2)	0.54957 (17)	0.0673 (14)	
H1	0.9131	0.2611	0.5180	0.081*	
C2	1.0297 (5)	0.2132 (3)	0.5532 (2)	0.0814 (18)	
H2	1.0658	0.1998	0.5246	0.098*	
C3	1.0669 (5)	0.1972 (2)	0.6000 (2)	0.0747 (16)	
H3	1.1305	0.1741	0.6035	0.090*	
C4	1.0095 (4)	0.2154 (2)	0.64189 (17)	0.0546 (11)	
H4	1.0326	0.2039	0.6737	0.066*	
C5	0.9172 (3)	0.25110 (17)	0.63568 (14)	0.0419 (9)	
C6	0.8486 (3)	0.27313 (16)	0.67749 (13)	0.0360 (8)	
C7	0.8605 (3)	0.24717 (17)	0.72905 (13)	0.0384 (8)	
C8	0.7886 (4)	0.2016 (2)	0.74534 (17)	0.0546 (11)	
H8	0.7321	0.1890	0.7247	0.066*	
C9	0.8002 (5)	0.1745 (2)	0.7924 (2)	0.0696 (14)	
H9	0.7519	0.1436	0.8032	0.084*	
C10	0.8835 (5)	0.1935 (3)	0.82286 (19)	0.0758 (16)	
H10	0.8909	0.1758	0.8546	0.091*	
C11	0.9552 (5)	0.2379 (3)	0.80682 (19)	0.0831 (17)	
H11	1.0119	0.2504	0.8275	0.100*	
C12	0.9440 (4)	0.2645 (2)	0.76020 (17)	0.0653 (13)	
H12	0.9937	0.2947	0.7494	0.078*	
C13	0.6370 (3)	0.37643 (18)	0.66655 (14)	0.0427 (9)	
C14	0.3281 (4)	−0.0096 (2)	0.44722 (15)	0.0527 (11)	
H14	0.3484	0.0040	0.4790	0.063*	
C15	0.2455 (4)	−0.0522 (3)	0.44297 (19)	0.0741 (16)	
H15	0.2098	−0.0671	0.4713	0.089*	
C16	0.2161 (5)	−0.0725 (3)	0.3959 (2)	0.0832 (18)	
H16	0.1602	−0.1016	0.3920	0.100*	
C17	0.2703 (4)	−0.0494 (2)	0.35449 (17)	0.0626 (13)	
H17	0.2513	−0.0627	0.3225	0.075*	
C18	0.3520 (3)	−0.00673 (18)	0.36126 (13)	0.0403 (8)	
C19	0.4168 (3)	0.02092 (17)	0.32052 (13)	0.0368 (8)	
C20	0.4045 (3)	−0.00038 (17)	0.26745 (13)	0.0369 (8)	
C21	0.3209 (4)	0.0214 (2)	0.23788 (15)	0.0562 (11)	
H21	0.2697	0.0492	0.2508	0.067*	
C22	0.3144 (4)	0.0011 (3)	0.18867 (17)	0.0664 (13)	
H22	0.2580	0.0154	0.1687	0.080*	
C23	0.3888 (4)	−0.0392 (2)	0.16898 (16)	0.0644 (14)	
H23	0.3844	−0.0514	0.1356	0.077*	
C24	0.4709 (4)	−0.0618 (2)	0.19901 (19)	0.0647 (14)	
H24	0.5207	−0.0906	0.1862	0.078*	
C25	0.4789 (4)	−0.0417 (2)	0.24776 (17)	0.0521 (10)	
H25	0.5354	−0.0562	0.2676	0.062*	
C26	0.6197 (3)	0.13029 (17)	0.33247 (14)	0.0409 (9)	
C27	0.5897 (4)	0.1987 (2)	0.56680 (14)	0.0471 (10)	
C28	0.5253 (3)	0.12554 (19)	0.51235 (14)	0.0447 (9)	
C29	0.7585 (3)	0.3768 (2)	0.48527 (16)	0.0483 (10)	
C30	0.8225 (3)	0.4478 (2)	0.42797 (13)	0.0455 (10)	

### Atomic displacement parameters (Å^2^)

**Table:**

	*U*^11^	*U*^22^	*U*^33^	*U*^12^	*U*^13^	*U*^23^
Cu1	0.0501 (3)	0.0411 (3)	0.0272 (2)	−0.00041 (19)	0.00001 (19)	−0.00053 (18)
Cu2	0.0476 (3)	0.0394 (3)	0.0245 (2)	−0.0023 (2)	−0.00390 (18)	−0.00204 (17)
O1	0.070 (2)	0.0597 (18)	0.0327 (14)	0.0195 (15)	−0.0050 (13)	0.0012 (12)
O2	0.0662 (19)	0.0471 (16)	0.0334 (14)	−0.0157 (14)	−0.0080 (13)	−0.0001 (11)
O1W	0.220 (9)	0.323 (11)	0.266 (10)	0.038 (8)	−0.025 (8)	−0.002 (8)
O2W	0.247 (10)	0.253 (11)	0.201 (8)	0.037 (8)	−0.069 (8)	−0.052 (8)
O1W'	0.220 (9)	0.323 (11)	0.266 (10)	0.038 (8)	−0.025 (8)	−0.002 (8)
O2W'	0.247 (10)	0.253 (11)	0.201 (8)	0.037 (8)	−0.069 (8)	−0.052 (8)
N1	0.059 (2)	0.0457 (18)	0.0354 (17)	0.0038 (16)	0.0107 (15)	0.0022 (14)
N2	0.0452 (18)	0.0354 (16)	0.0281 (14)	0.0019 (14)	−0.0007 (13)	−0.0020 (12)
N3	0.046 (2)	0.0472 (18)	0.0323 (16)	0.0081 (15)	−0.0028 (14)	−0.0045 (14)
N4	0.075 (3)	0.078 (3)	0.0401 (19)	0.036 (2)	0.0021 (18)	0.0022 (18)
N5	0.0458 (19)	0.0448 (17)	0.0282 (15)	−0.0011 (14)	0.0010 (13)	0.0004 (13)
N6	0.0418 (18)	0.0395 (17)	0.0261 (14)	−0.0010 (13)	−0.0031 (12)	−0.0002 (12)
N7	0.0487 (19)	0.0429 (17)	0.0266 (14)	−0.0060 (15)	−0.0046 (13)	0.0028 (13)
N8	0.070 (3)	0.054 (2)	0.0427 (19)	−0.0245 (19)	−0.0014 (17)	0.0026 (16)
N9	0.090 (3)	0.063 (2)	0.052 (2)	−0.027 (2)	0.001 (2)	−0.0150 (19)
N10	0.097 (3)	0.082 (3)	0.041 (2)	−0.045 (3)	0.021 (2)	−0.029 (2)
N11	0.062 (2)	0.055 (2)	0.0321 (17)	−0.0067 (17)	−0.0025 (15)	−0.0054 (15)
N12	0.058 (2)	0.056 (2)	0.0389 (19)	−0.0099 (17)	0.0001 (16)	0.0063 (16)
N13	0.100 (3)	0.096 (3)	0.043 (2)	−0.056 (3)	−0.028 (2)	0.030 (2)
N14	0.061 (2)	0.058 (2)	0.0425 (19)	−0.0145 (19)	−0.0045 (17)	0.0155 (16)
C1	0.089 (4)	0.068 (3)	0.044 (2)	0.022 (3)	0.027 (2)	0.007 (2)
C2	0.101 (4)	0.084 (4)	0.060 (3)	0.041 (3)	0.036 (3)	0.007 (3)
C3	0.079 (4)	0.069 (3)	0.076 (4)	0.035 (3)	0.031 (3)	0.011 (3)
C4	0.062 (3)	0.051 (2)	0.051 (3)	0.012 (2)	0.007 (2)	0.009 (2)
C5	0.050 (2)	0.0348 (19)	0.041 (2)	0.0007 (17)	0.0088 (17)	0.0028 (15)
C6	0.043 (2)	0.0308 (17)	0.0345 (18)	−0.0025 (16)	0.0010 (15)	0.0002 (14)
C7	0.043 (2)	0.0394 (19)	0.0326 (18)	0.0044 (16)	0.0035 (16)	0.0042 (15)
C8	0.062 (3)	0.047 (2)	0.056 (3)	−0.007 (2)	0.004 (2)	0.005 (2)
C9	0.079 (4)	0.056 (3)	0.074 (3)	−0.002 (3)	0.023 (3)	0.020 (3)
C10	0.088 (4)	0.089 (4)	0.051 (3)	0.012 (3)	0.002 (3)	0.034 (3)
C11	0.081 (4)	0.121 (5)	0.048 (3)	−0.013 (4)	−0.019 (3)	0.025 (3)
C12	0.064 (3)	0.085 (3)	0.047 (2)	−0.025 (3)	−0.011 (2)	0.016 (2)
C13	0.051 (2)	0.042 (2)	0.035 (2)	0.0092 (18)	−0.0012 (17)	−0.0034 (16)
C14	0.059 (3)	0.065 (3)	0.033 (2)	−0.002 (2)	0.0065 (19)	0.0007 (18)
C15	0.066 (3)	0.106 (4)	0.051 (3)	−0.023 (3)	0.011 (2)	0.014 (3)
C16	0.071 (4)	0.118 (5)	0.061 (3)	−0.043 (3)	−0.003 (3)	0.013 (3)
C17	0.064 (3)	0.082 (3)	0.042 (2)	−0.032 (3)	−0.006 (2)	0.003 (2)
C18	0.042 (2)	0.048 (2)	0.0305 (18)	−0.0046 (17)	−0.0027 (15)	−0.0003 (15)
C19	0.042 (2)	0.0369 (19)	0.0314 (18)	−0.0001 (16)	−0.0041 (15)	0.0008 (14)
C20	0.042 (2)	0.0375 (19)	0.0307 (18)	−0.0070 (16)	−0.0029 (15)	−0.0030 (14)
C21	0.059 (3)	0.071 (3)	0.038 (2)	0.011 (2)	−0.011 (2)	−0.008 (2)
C22	0.076 (3)	0.084 (3)	0.039 (2)	−0.002 (3)	−0.023 (2)	−0.008 (2)
C23	0.086 (4)	0.074 (3)	0.034 (2)	−0.028 (3)	0.004 (2)	−0.019 (2)
C24	0.071 (3)	0.055 (3)	0.069 (3)	−0.007 (2)	0.020 (3)	−0.032 (2)
C25	0.053 (3)	0.054 (3)	0.049 (2)	−0.001 (2)	−0.003 (2)	−0.007 (2)
C26	0.050 (2)	0.037 (2)	0.0356 (19)	0.0002 (17)	−0.0006 (17)	0.0036 (16)
C27	0.060 (3)	0.053 (2)	0.0280 (18)	−0.007 (2)	0.0055 (18)	−0.0048 (17)
C28	0.052 (2)	0.049 (2)	0.033 (2)	−0.0117 (19)	0.0037 (17)	−0.0062 (17)
C29	0.047 (2)	0.054 (2)	0.043 (2)	−0.0148 (19)	−0.0113 (18)	0.0046 (19)
C30	0.050 (2)	0.062 (3)	0.0252 (18)	−0.008 (2)	−0.0133 (17)	0.0066 (17)

### Geometric parameters (Å, º)

**Table:**

Cu1—N2	1.946 (3)	C1—H1	0.9300
Cu1—N12	1.955 (3)	C2—C3	1.376 (7)
Cu1—O1	1.966 (3)	C2—H2	0.9300
Cu1—N1	2.004 (4)	C3—C4	1.380 (6)
Cu1—N9	2.205 (4)	C3—H3	0.9300
Cu2—N6	1.940 (3)	C4—C5	1.377 (6)
Cu2—N11	1.941 (3)	C4—H4	0.9300
Cu2—O2	1.977 (3)	C5—C6	1.479 (5)
Cu2—N5	2.016 (3)	C6—C7	1.488 (5)
Cu2—N14^i^	2.228 (4)	C7—C8	1.378 (6)
O1—C13	1.278 (4)	C7—C12	1.378 (6)
O2—C26	1.281 (4)	C8—C9	1.387 (7)
O1W—H1W1	0.8401	C8—H8	0.9300
O1W—H1W2	0.8401	C9—C10	1.375 (8)
O2W—H2W1	0.8400	C9—H9	0.9300
O2W—H2W2	0.8400	C10—C11	1.359 (8)
N1—C1	1.333 (5)	C10—H10	0.9300
N1—C5	1.346 (5)	C11—C12	1.371 (6)
N2—C6	1.292 (5)	C11—H11	0.9300
N2—N3	1.358 (4)	C12—H12	0.9300
N3—C13	1.349 (5)	C14—C15	1.364 (7)
N4—C13	1.334 (5)	C14—H14	0.9300
N4—H41	0.8800	C15—C16	1.377 (7)
N4—H42	0.8800	C15—H15	0.9300
N5—C14	1.331 (5)	C16—C17	1.383 (7)
N5—C18	1.356 (4)	C16—H16	0.9300
N6—C19	1.284 (5)	C17—C18	1.365 (6)
N6—N7	1.358 (4)	C17—H17	0.9300
N7—C26	1.353 (5)	C18—C19	1.472 (5)
N8—C26	1.332 (5)	C19—C20	1.494 (5)
N8—H81	0.8800	C20—C25	1.372 (6)
N8—H82	0.8800	C20—C21	1.381 (6)
N9—C27	1.129 (5)	C21—C22	1.384 (6)
N10—C28	1.280 (5)	C21—H21	0.9300
N10—C27	1.296 (6)	C22—C23	1.358 (7)
N11—C28	1.143 (5)	C22—H22	0.9300
N12—C29	1.127 (5)	C23—C24	1.381 (7)
N13—C29	1.300 (5)	C23—H23	0.9300
N13—C30	1.303 (6)	C24—C25	1.372 (6)
N14—C30	1.127 (5)	C24—H24	0.9300
N14—Cu2^ii^	2.228 (4)	C25—H25	0.9300
C1—C2	1.372 (7)		
			
N2—Cu1—N12	163.76 (14)	N2—C6—C7	125.0 (3)
N2—Cu1—O1	79.14 (12)	C5—C6—C7	121.8 (3)
N12—Cu1—O1	97.91 (14)	C8—C7—C12	118.6 (4)
N2—Cu1—N1	80.51 (13)	C8—C7—C6	118.8 (4)
N12—Cu1—N1	99.07 (14)	C12—C7—C6	122.4 (4)
O1—Cu1—N1	157.69 (13)	C7—C8—C9	120.3 (5)
N2—Cu1—N9	98.30 (14)	C7—C8—H8	119.9
N12—Cu1—N9	97.91 (15)	C9—C8—H8	119.9
O1—Cu1—N9	97.52 (15)	C10—C9—C8	119.6 (5)
N1—Cu1—N9	94.37 (16)	C10—C9—H9	120.2
N6—Cu2—N11	165.22 (14)	C8—C9—H9	120.2
N6—Cu2—O2	79.29 (12)	C11—C10—C9	120.3 (4)
N11—Cu2—O2	96.58 (13)	C11—C10—H10	119.8
N6—Cu2—N5	80.58 (12)	C9—C10—H10	119.8
N11—Cu2—N5	100.68 (13)	C10—C11—C12	120.0 (5)
O2—Cu2—N5	158.01 (11)	C10—C11—H11	120.0
N6—Cu2—N14^i^	96.54 (13)	C12—C11—H11	120.0
N11—Cu2—N14^i^	98.04 (14)	C11—C12—C7	121.1 (4)
O2—Cu2—N14^i^	96.66 (13)	C11—C12—H12	119.5
N5—Cu2—N14^i^	94.34 (14)	C7—C12—H12	119.5
C13—O1—Cu1	110.4 (2)	O1—C13—N4	118.9 (4)
C26—O2—Cu2	110.4 (2)	O1—C13—N3	125.2 (3)
H1W1—O1W—H1W2	108.7	N4—C13—N3	115.9 (3)
H2W1—O2W—H2W2	107.9	N5—C14—C15	122.6 (4)
C1—N1—C5	119.4 (4)	N5—C14—H14	118.7
C1—N1—Cu1	127.6 (3)	C15—C14—H14	118.7
C5—N1—Cu1	112.9 (3)	C14—C15—C16	118.6 (4)
C6—N2—N3	124.0 (3)	C14—C15—H15	120.7
C6—N2—Cu1	117.9 (2)	C16—C15—H15	120.7
N3—N2—Cu1	117.5 (2)	C15—C16—C17	119.5 (5)
C13—N3—N2	106.8 (3)	C15—C16—H16	120.3
C13—N4—H41	120.0	C17—C16—H16	120.3
C13—N4—H42	120.0	C18—C17—C16	119.1 (4)
H41—N4—H42	120.0	C18—C17—H17	120.5
C14—N5—C18	119.1 (4)	C16—C17—H17	120.5
C14—N5—Cu2	128.3 (3)	N5—C18—C17	121.2 (4)
C18—N5—Cu2	112.3 (2)	N5—C18—C19	114.2 (3)
C19—N6—N7	124.2 (3)	C17—C18—C19	124.5 (3)
C19—N6—Cu2	117.7 (2)	N6—C19—C18	114.3 (3)
N7—N6—Cu2	117.7 (2)	N6—C19—C20	123.7 (3)
C26—N7—N6	107.4 (3)	C18—C19—C20	121.8 (3)
C26—N8—H81	120.0	C25—C20—C21	119.7 (4)
C26—N8—H82	120.0	C25—C20—C19	119.0 (3)
H81—N8—H82	120.0	C21—C20—C19	121.4 (3)
C27—N9—Cu1	168.1 (4)	C20—C21—C22	119.0 (4)
C28—N10—C27	122.9 (4)	C20—C21—H21	120.5
C28—N11—Cu2	166.7 (3)	C22—C21—H21	120.5
C29—N12—Cu1	170.9 (4)	C23—C22—C21	121.4 (5)
C29—N13—C30	122.2 (4)	C23—C22—H22	119.3
C30—N14—Cu2^ii^	164.2 (3)	C21—C22—H22	119.3
N1—C1—C2	122.2 (5)	C22—C23—C24	119.4 (4)
N1—C1—H1	118.9	C22—C23—H23	120.3
C2—C1—H1	118.9	C24—C23—H23	120.3
C1—C2—C3	118.5 (4)	C25—C24—C23	119.9 (4)
C1—C2—H2	120.7	C25—C24—H24	120.1
C3—C2—H2	120.7	C23—C24—H24	120.1
C2—C3—C4	119.7 (5)	C20—C25—C24	120.7 (4)
C2—C3—H3	120.1	C20—C25—H25	119.7
C4—C3—H3	120.1	C24—C25—H25	119.7
C5—C4—C3	118.8 (4)	O2—C26—N8	119.4 (4)
C5—C4—H4	120.6	O2—C26—N7	124.6 (3)
C3—C4—H4	120.6	N8—C26—N7	116.0 (3)
N1—C5—C4	121.2 (4)	N9—C27—N10	173.3 (5)
N1—C5—C6	114.9 (3)	N11—C28—N10	172.7 (5)
C4—C5—C6	123.9 (4)	N12—C29—N13	173.4 (5)
N2—C6—C5	112.9 (3)	N14—C30—N13	174.2 (4)
			
N2—Cu1—O1—C13	−7.3 (3)	C3—C4—C5—N1	−0.8 (7)
N12—Cu1—O1—C13	−171.1 (3)	C3—C4—C5—C6	179.6 (4)
N1—Cu1—O1—C13	−31.8 (5)	N3—N2—C6—C5	−178.4 (3)
N9—Cu1—O1—C13	89.8 (3)	Cu1—N2—C6—C5	10.5 (4)
N6—Cu2—O2—C26	−5.6 (3)	N3—N2—C6—C7	7.6 (6)
N11—Cu2—O2—C26	−171.2 (3)	Cu1—N2—C6—C7	−163.6 (3)
N5—Cu2—O2—C26	−29.6 (5)	N1—C5—C6—N2	−7.2 (5)
N14^i^—Cu2—O2—C26	89.9 (3)	C4—C5—C6—N2	172.5 (4)
N2—Cu1—N1—C1	−178.0 (4)	N1—C5—C6—C7	167.1 (3)
N12—Cu1—N1—C1	−14.5 (4)	C4—C5—C6—C7	−13.2 (6)
O1—Cu1—N1—C1	−153.6 (4)	N2—C6—C7—C8	72.8 (5)
N9—Cu1—N1—C1	84.3 (4)	C5—C6—C7—C8	−100.8 (5)
N2—Cu1—N1—C5	3.5 (3)	N2—C6—C7—C12	−110.7 (5)
N12—Cu1—N1—C5	167.1 (3)	C5—C6—C7—C12	75.7 (5)
O1—Cu1—N1—C5	28.0 (5)	C12—C7—C8—C9	0.6 (7)
N9—Cu1—N1—C5	−94.2 (3)	C6—C7—C8—C9	177.3 (4)
N12—Cu1—N2—C6	−98.0 (5)	C7—C8—C9—C10	0.3 (7)
O1—Cu1—N2—C6	−178.9 (3)	C8—C9—C10—C11	−0.9 (9)
N1—Cu1—N2—C6	−8.1 (3)	C9—C10—C11—C12	0.6 (9)
N9—Cu1—N2—C6	84.9 (3)	C10—C11—C12—C7	0.4 (9)
N12—Cu1—N2—N3	90.3 (6)	C8—C7—C12—C11	−1.0 (8)
O1—Cu1—N2—N3	9.4 (3)	C6—C7—C12—C11	−177.5 (5)
N1—Cu1—N2—N3	−179.8 (3)	Cu1—O1—C13—N4	−175.2 (3)
N9—Cu1—N2—N3	−86.8 (3)	Cu1—O1—C13—N3	5.1 (5)
C6—N2—N3—C13	180.0 (3)	N2—N3—C13—O1	2.2 (5)
Cu1—N2—N3—C13	−8.9 (4)	N2—N3—C13—N4	−177.5 (4)
N6—Cu2—N5—C14	−179.3 (4)	C18—N5—C14—C15	−0.4 (7)
N11—Cu2—N5—C14	−14.2 (4)	Cu2—N5—C14—C15	−173.8 (4)
O2—Cu2—N5—C14	−155.3 (3)	N5—C14—C15—C16	0.4 (9)
N14^i^—Cu2—N5—C14	84.8 (4)	C14—C15—C16—C17	−0.2 (10)
N6—Cu2—N5—C18	6.9 (3)	C15—C16—C17—C18	0.0 (9)
N11—Cu2—N5—C18	172.0 (3)	C14—N5—C18—C17	0.2 (6)
O2—Cu2—N5—C18	30.9 (5)	Cu2—N5—C18—C17	174.7 (4)
N14^i^—Cu2—N5—C18	−89.0 (3)	C14—N5—C18—C19	−179.1 (4)
N11—Cu2—N6—C19	−104.8 (6)	Cu2—N5—C18—C19	−4.7 (4)
O2—Cu2—N6—C19	−179.8 (3)	C16—C17—C18—N5	0.0 (8)
N5—Cu2—N6—C19	−8.7 (3)	C16—C17—C18—C19	179.2 (5)
N14^i^—Cu2—N6—C19	84.7 (3)	N7—N6—C19—C18	−178.9 (3)
N11—Cu2—N6—N7	82.0 (6)	Cu2—N6—C19—C18	8.4 (4)
O2—Cu2—N6—N7	7.0 (2)	N7—N6—C19—C20	5.5 (6)
N5—Cu2—N6—N7	178.1 (3)	Cu2—N6—C19—C20	−167.3 (3)
N14^i^—Cu2—N6—N7	−88.5 (3)	N5—C18—C19—N6	−2.1 (5)
C19—N6—N7—C26	−179.3 (3)	C17—C18—C19—N6	178.6 (4)
Cu2—N6—N7—C26	−6.6 (4)	N5—C18—C19—C20	173.7 (3)
N2—Cu1—N9—C27	−157 (2)	C17—C18—C19—C20	−5.7 (6)
N12—Cu1—N9—C27	24 (2)	N6—C19—C20—C25	74.0 (5)
O1—Cu1—N9—C27	123 (2)	C18—C19—C20—C25	−101.3 (4)
N1—Cu1—N9—C27	−76 (2)	N6—C19—C20—C21	−105.0 (5)
N6—Cu2—N11—C28	−52.0 (19)	C18—C19—C20—C21	79.7 (5)
O2—Cu2—N11—C28	20.8 (16)	C25—C20—C21—C22	−0.3 (7)
N5—Cu2—N11—C28	−145.5 (16)	C19—C20—C21—C22	178.7 (4)
N14^i^—Cu2—N11—C28	118.5 (16)	C20—C21—C22—C23	−0.5 (8)
C5—N1—C1—C2	−1.6 (8)	C21—C22—C23—C24	1.8 (8)
Cu1—N1—C1—C2	−179.9 (4)	C22—C23—C24—C25	−2.3 (7)
N1—C1—C2—C3	−1.0 (9)	C21—C20—C25—C24	−0.3 (6)
C1—C2—C3—C4	2.7 (9)	C19—C20—C25—C24	−179.3 (4)
C2—C3—C4—C5	−1.8 (8)	C23—C24—C25—C20	1.6 (7)
C1—N1—C5—C4	2.5 (6)	Cu2—O2—C26—N8	−175.3 (3)
Cu1—N1—C5—C4	−179.0 (3)	Cu2—O2—C26—N7	4.0 (5)
C1—N1—C5—C6	−177.8 (4)	N6—N7—C26—O2	1.5 (5)
Cu1—N1—C5—C6	0.7 (4)	N6—N7—C26—N8	−179.2 (3)

Symmetry codes: (i) −*x*+3/2, *y*−1/2, *z*; (ii) −*x*+3/2, *y*+1/2, *z*.

### Hydrogen-bond geometry (Å, º)

**Table:**

*D*—H···*A*	*D*—H	H···*A*	*D*···*A*	*D*—H···*A*
N4—H42···N7^iii^	0.88	2.13	3.006 (5)	176
N8—H82···N3^iv^	0.88	2.15	3.025 (5)	179
O1w—H1w1···O1	0.84	2.05	2.88 (2)	169
O2w—H2w1···O2	0.84	2.34	3.151 (19)	161

Symmetry codes: (iii) *x*, −*y*+1/2, *z*+1/2; (iv) *x*, −*y*+1/2, *z*−1/2.
